# The History of the British Officers' Library, Stralsund, Germany

**Published:** 1920-08-14

**Authors:** 


### A PRISONERS OF WAR LIBRARY.

### The History of the British Officers' Library, Stralsund, Germany.

It is very difficult to convey to the mind of anyone who has not lived in a prisoners of war camp any idea of the numerous difficulties one has there to contend with in organising a library or any other branch of camp life. For the first three months of our captivity, before we received our food-parcels from England, we were practically starved, and everyone was so extraordinarily weak that very often the walk of some 300 yards from our barrack to the library was sufficient to incapacitate us for the rest of the day. We found that two hours' work daily was as much as we could do. This in itself made progress very slow.

### The Need for Books.

Both the writers of this article (one the librarian of a London hospital) were captured on the Chemin-des-Dames on May 27, 1918. A fortnight later we reached a temporary camp at Rastatt, where there were some 400 British and several hundred French officers. Apart from eating the very small quantity of excessively unappetising food which we were given by the German Government, there was very little to be done all day. We had no facilities for doing any kind of work or playing any games, so that time hung very heavily on our hands. Everyone felt the need for something to read—even if that something was only a copy of the Army List or Bradshaw's Railway Guide. The only available reading material was a few volumes of the Tauchnitz edition on sale at the camp canteen. Had these books been some of the best of the many excellent volumes published in the Tauchnitz edition they would have been very welcome; but, unfortunately, they were, almost without exception, a collection of the worst and most uninteresting in the whole series. Also the canteen price was excessive, being 3 marks 50 for a volume published at 1 mark 60. As very few of us had any money—any we originally had having been "borrowed" by the enemy on our capture—it was difficult to buy these books. When it was possible to cash a cheque—the German authorities decided that the mark equalled a shilling, whereas the present rate of exchange shows how much we lost over the deal. It was soon apparent that there was a great need for some kind of organised library; at the same time it was obvious that it was no use attempting to start one until we reached a permanent camp, Rastatt being only a distributing station where everyone was liable to depart at a few hours' notice for another camp.

The only possible way for officers to obtain a variety of literature was by exchanging with one another the few books they had been able to purchase at the canteen. The books, all of which were unbound, soon became very dilapidated, and had a habit of losing some of their pages, usually towards the end of the book, so that the wretched reader did not discover their absence until he had read the bulk of the book. Many of the books were in two or more volumes, but you were a lucky man if you ever saw more than one volume of a work.

On June 27 we formed part of a batch of four hundred officers who were removed to a new permanent camp on the Island of Danholm, near Stralsund.

Our preliminary difficulties were numerous. In the first place, we had considerable trouble in inducing the German authorities to allot us a room for the library, but eventually they gave us a room which had formerly been a canteen—care being taken to remove all the beer before the Library Committee entered it! This room was an ideal one for the purpose. It contained a large bay, which we used as a book store and circulating department, the rest being set aside as a reading-room.

We had some difficulty in arranging suitable hours for the library to be open. During most of the afternoon the room was wanted by the Education Committee for some of their classes. Then the hours of meals had to be studied in order to allow the members on duty to obtain their small portion of cabbage soup, which formed the main article of our diet. As the meals worked in three shifts, they took up much of the day. It soon became evident that the hours of circulation would have to be increased, as day after day for the first week or so of the library's existence there were long queues of more or less—mostly less—patient members waiting to exchange books. As the days became shorter, this difficulty was increased, about half the officers being not allowed to go across to that part of the camp where the library was situated after dark, so it was impossible to open the library in the evenings. This considerably reduced the hours of circulation.

### The Opening of the Library.

The opening of the library was delayed for over a fortnight, on account of the Camp Commandant ordering all officers to be "deloused," whether they required that treatment or not. The inhabitants of each barrack went in turn to the delousing station on the other side of the island, where the delousing process took three days. This necessitated a wait until the last barrack had been done before we could call in the books that were in the camp, the nucleus of our library. The library was finally opened on July 24, 1918, with a membership of 467 and a stock of 993 books, of which 528 were English, 217 French, and 248 German. Of this total, all the foreign works and ninety of the English books were lent by the German authorities. These volumes formed part of a Camp Library collected by Russian officers who were once prisoners of war on our island. Of the remainder of the English books, sixty had been presented by the Berlin Y.M.C.A., and the balance had been given by officers in the camp, as part of their entrance fees. A library of 528 English volumes sounds quite a respectable one, but many of the books were odd volumes or had pages missing, or, if they happened to have escaped these drawbacks, their subject-matter was often of a very "trashy" description, so that the actual reading material was lamentably small. A few days before the opening was due we bumped up against an unexpected trouble by some of the German Guard marching into the room, pitching us out, locking the door, and taking away the only key we had. It took us a couple of days to get this back again. We balanced accounts on this point by finding another key of the room, which, we regret to say, we stole without the slightest hesitation or qualms of conscience: a sad example of the depths to which even a librarian may sink under certain circumstances.

We endeavoured to improve the standard by introducing

**A Prisoners of War Library**—(*continued*).

more books by well-known authors and works of an educational character, at the same time casting out some of the trashy novels presented in the early days of the library, when we were only too glad to extend the heartiest of welcomes to anything that had ever seen a printer's press. We obtained through the Prisoners of War Book Scheme many hundreds of volumes of an educational and technical nature. These books were issued on permanent loan to men pursuing their professional studies. Many of these volumes were the principal text-books on the various subjects, and their value to the camp as a whole cannot be overestimated. The greatest praise is due to Sir Alfred Davies and other promoters of this scheme, which was practically the only available source of supply open to men wishing to continue or commence their professional studies. Most of the books were used in conjunction with the many excellent classes organised by our Camp Education Committee. We obtained some 150 grammars of various European languages, which were supplied at a very low price by the Berlin Y.M.C.A. But our greatest difficulty—we had many—was the acquisition of more general literature. Practically the only books we could purchase were those published in the Tauchnitz edition. As we have already stated, we started with a total of 993 volumes, which number we endeavoured to augment as rapidly as possible. We asked every officer in the camp to present to the library any books he might receive from time to time, and we ordered 200 volumes of the Tauchnitz edition through a German bookseller who visited the camp for orders. We had to pay 5 marks per volume for these! There was a civilian clerk in camp from whom we purchased 159 volumes for 262 marks, and when we went to pay him he refused to take cash, imploring us to let him have food instead as soon as our food parcels arrived. The fact that a man in his position, with a wife and children to support, was willing to sacrifice so large a sum as £13 for an uncertain amount of food at some indefinite date in the future shows how greatly the Germans were in need of food at that time.

In addition to these books, we obtained two splendid donations, one of 500 volumes of the Tauchnitz edition from the Danish Red Cross and one of 255 similar volumes from the Berlin Y.M.C.A., as well as many more foreign works on loan from the German authorities. Later, in these and other smaller ways we gradually increased our library, until, when it was finally disbanded after the armistice, it contained 1,893 English books, 311 French, and 372 German books.

### Book-binding.

We had a room allotted for binding purposes; but it was of very little use, as we could not procure the necessary materials. Whenever we asked the commandant to assist us in obtaining these materials—for which we were willing to pay—we were always met by the stock answer: "That they were unable to obtain them owing to the efficiency of our blockade," a cheery if unsatisfactory explanation. All books before being handed over were censored by the camp censor. Fortunately this censoring was more or less an eyewash, as several books trickled through containing sentiments of anything but a pro-German nature. Early in October the Germans adopted the practice of stripping the binding off all books that came into the camp. The reason for this was that a book had been discovered containing maps and German notes of considerable value concealed in the binding.

When the library closed at the end of November it contained 2,576 volumes, apart from those issued to members under the Prisoners of War Book Scheme, and had a membership of 1,174. These volumes, with the exception of those lent by the German authorities, were distributed among the members when the library was closed, and so came to an end what was, in all probability, the largest library of its kind in the world.

